# An Integrated Dual-Layer Heterogeneous Polycaprolactone Scaffold Promotes Oral Mucosal Wound Healing through Inhibiting Bacterial Adhesion and Mediating HGF-1 Behavior

**DOI:** 10.34133/research.0499

**Published:** 2024-10-24

**Authors:** Gaoying Hong, Zihe Hu, Yanyan Zhou, Mumian Chen, Haiyan Wu, Weiying Lu, Wenjing Jin, Ke Yao, Zhijian Xie, Jue Shi

**Affiliations:** ^1^ Stomatology Hospital, School of Stomatology, Zhejiang University School of Medicine, Zhejiang Provincial Clinical Research Center for Oral Diseases, Key Laboratory of Oral Biomedical Research of Zhejiang Province, Cancer Center of Zhejiang University, Engineering Research Center of Oral Biomaterials and Devices of Zhejiang Province, Hangzhou 310000, China.; ^2^ State Key Laboratory of Fluid Power and Mechatronic Systems, School of Mechanical Engineering, Zhejiang University, Hangzhou 310027, China.

## Abstract

Recently, the high incidence of oral mucosal defects and the subsequent functional impairments have attracted widespread attention. Controlling scaffold geometry pattern has been proposed as a strategy to promote cell behavior and facilitate soft tissue repair. In this study, we innovatively construct an integrated dual-layer heterogeneous polycaprolactone (PCL) scaffold using melt electrowriting (MEW) technology. The outer layer was disordered, while the inner layer featured oriented fiber patterns: parallel (P-par), rhombic (P-rhomb), and square (P-sq). Our findings revealed that the P-rhomb and P-sq scaffolds exhibited superior surface wettability, roughness, and tensile strength compared to the pure disordered PCL scaffolds (P) and P-par. Compared to the commercial collagen membranes, the outer layer of PCL can effectively inhibit bacterial adhesion and biofilm formation. Furthermore, the P-rhomb and P-sq groups demonstrated higher gene and protein expression levels related to cell adhesion and cell migration rates than did the P and P-par groups. Among them, P-sq plays an important role in inducing the differentiation of gingival fibroblasts into myofibroblasts rich in α-smooth muscle actin (α-SMA). Additionally, P-sq could reduce inflammation, promote epithelial regeneration, and accelerate wound healing when used in full-thickness oral mucosal defects in rabbits. Overall, the integrated dual-layer heterogeneous PCL scaffold fabricated by MEW technology effectively inhibited bacterial adhesion and guided tissue regeneration, offering advantages for clinical translation and large-scale production. This promising material holds important potential for treating full-thickness mucosal defects in a bacteria-rich oral environments.

## Introduction

The oral mucosa serves as the primary defense against various harmful substances, safeguarding the underlying mucosal tissues. Soft tissue defects caused by tooth extraction, surgical resection, trauma, and oral mucosal diseases are among the most common clinical conditions. Improper treatment can lead to morphological abnormalities, functional impairments, and a decreased quality of life for patients. In more severe cases, it can even cause inflammation and necrosis of deep tissue organs [1]. Currently, autologous skin or flap transplantation are the most commonly employed methods for repairing oral mucosal defects [2,3]. While effective, this approach necessitates sacrificing normal tissue and causes secondary trauma. Consequently, developing an ideal biomaterial for reconstructing oral mucosal defects holds great importance.

In recent years, researchers have developed a series of oral mucosal repair materials with antibacterial properties or the ability to regulate macrophage anti-inflammatory responses [4,5]. It is worth noting that despite the importance of improving the immune microenvironment of the wound, the important role of gingival fibroblasts in the early stage of wound healing is often underestimated. It is well established that fibroblasts undergo a multitude of structural and functional modifications during the process of wound healing. These include the differentiation of fibroblasts into myofibroblasts, the formation of granulation tissue to fill the wound, the deposition and remodeling of the extracellular matrix (ECM), and their involvement in immune regulation [6]. Among these, the transient activation of fibroblasts into myofibroblasts is particularly beneficial for acute tissue injury repair due to their strong contractility and ability to facilitate rapid wound closure [7]. Consequently, creating a biomembrane capable of stimulating gingival myofibroblast activation and promoting swift wound healing is a promising avenue of research.

The interaction between materials and cells has been a major focus in tissue engineering and regenerative medicine research [8,9]. Beyond the surface’s biochemical properties, the material’s pattern structure, including macroscopic, microscopic, and nanoscale features, plays a crucial role in guiding cell behavior within the cellular microenvironment [10,11]. The parallel structure is one of the most widely studied patterned structures, which has been applied in bone regeneration, nerve repair, skin regeneration, and other fields. Studies have demonstrated that electrospun membranes with aligned structures influence the alignment of endothelial cells and the elongation of the body, thereby promoting cell migration and angiogenic differentiation, in comparison to conventional electrospun scaffolds with randomly deposited nanofibers [12,13]. Square pores have been found to enhance chondrogenic differentiation, whereas rhombus-shaped pores promote osteogenic differentiation in fused deposition modeling scaffolds [14]. These findings suggest that patterned structures can positively influence the biological behavior of cells. However, the specific impact of square and rhombus patterns on the biological behavior of gingival fibroblasts remains unexplored. Investigating whether scaffolds with patterned structures can facilitate oral mucosal wound healing would be valuable.

To generate scaffolds with specific geometric patterns, various fabrication techniques have been employed [15]. In particular, the application of electrospun meshes in soft tissue engineering has been extensively investigated, including skin, nerve, and tendon [16,17]. However, due to technical limitations, electrospinning cannot produce scaffolds with precisely oriented fibers and specific geometric patterns [15,18]. Recently, a substantial advancement in fibrous scaffold fabrication has been demonstrated with the ability to create integrated heterogeneous fibrous structures using melt electrowriting (MEW). MEW is a high-precision additive manufacturing technology that enables the precise deposition of single fibers and the production of ordered fiber-patterned structures. Moreover, melt direct writing eliminates the use of solvents, thereby avoiding the risk of solvent residue, making it particularly suitable for the preparation of biological materials [19].

In this study, we employed MEW technology to construct integrated dual-layer heterogeneous PCL scaffolds. The dense disordered outer layer serves as a barrier against bacterial invasion in the oral environment, while the patterned inner layer acts as a barrier and promotes early-stage soft tissue healing. Based on the idea of studying the effect of the geometry of intersecting fibers on the behavior of cells, we designed 3 fiber pattern structures: parallel (P-par), rhombic (P-rhomb), and square (P-sq) (where fiber crossing angles of fibers were 0°, 45°, and 90°, respectively). This study analyzed the different cellular behaviors of human gingival fibroblasts (HGF-1) on the surfaces of the 3 fiber-pattern structures in vitro and found that the square structure had the most optimal effect in promoting cell adhesion and migration and inducing HGF-1 to differentiate into gingival myofibroblasts rich in α-smooth muscle actin (α-SMA). Finally, we implanted scaffolds with the square structure into rabbit oral full-thickness soft tissue defects. Gross specimen analysis and immunohistochemical (IHC) staining confirmed the PCL scaffolds effectively inhibit wound inflammation, promote vascularization, and facilitate tissue repair. We envision that the integrated dual-layer PCL scaffold with specific fiber-pattern structures can be clinically transformed and large-scale produced, which has great clinical prospects in the field of oral mucosa repair.

## Results and Discussion

### Morphology and characterization of the PCL scaffolds

MEW technology combines melt electrospinning and 3-dimensional (3D) printing, enabling programmable control of fiber diameter and shape, thus achieving high controllability of the fiber structure [20]. This allows for the design of complex or geometric shapes under computer control. In a contaminated oral environment, the wound dressing should act as a barrier layer to isolate bacteria, maintaining a favorable microenvironment for tissue healing [21]. Simultaneously, the dressing should promote cellular adhesion and proliferation to facilitate expedited wound healing. The construction of patterned surfaces of biomaterials to guide the biological behavior of cells has become a recent research focus. Therefore, we first printed the disordered dense structure as the outer layer by MEW technology. Afterward, we constructed 3 specific patterns of ordered fibrous structures as the second layer: P-par, P-rhomb, and P-sq.

**Fig. 1.**
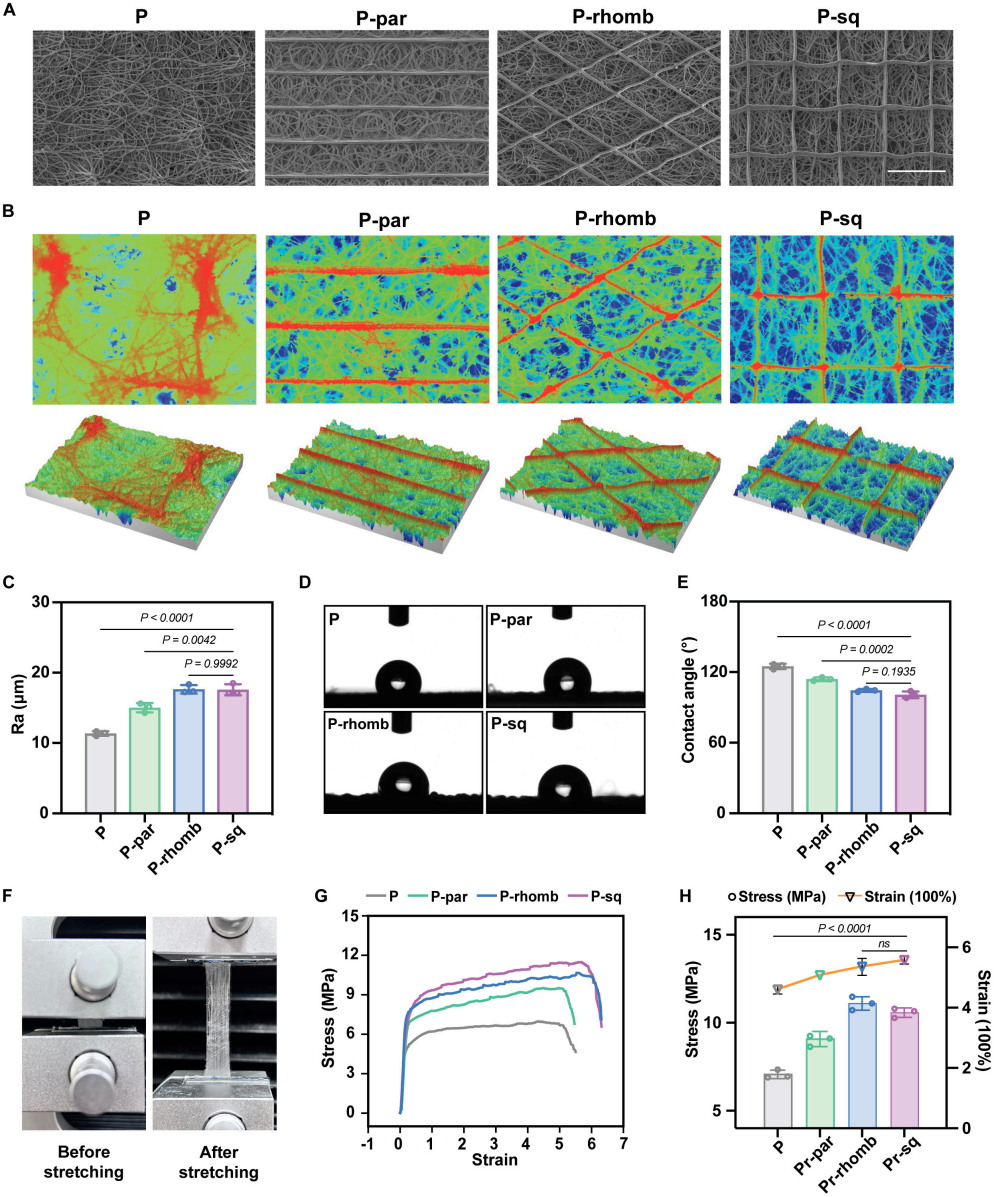
Morphology and characterization of the PCL scaffolds. (A) SEM images of the monolayer disordered PCL (P) and the dual-layer PCL (P-par, P-rhomb, P-sq). Scale bar, 250 μm. (B) Microprofilometer images. (C) Roughness analysis. (D) Representative images of contact angle test. (E) Water contact angle analysis. (F) Photos of tensile test. (G) Typical tensile stress–strain curves of P, P-par, P-rhomb, and P-sq. (H) Maximum tensile strength and corresponding strain of P, P-par, P-rhomb, and P-sq. All data are presented as means ± SD; *n* = 3.

First, we observed the outer surface of the disordered layer using scanning electron microscopy (SEM). When the PCL solution contacted the glass plate heated to 30 °C, it exhibited a certain degree of flowability, resulting in flattened fibers, while the inner surface of the disordered layer showed distinct “thread-like” fibers and appeared relatively rough (Fig. S1). Then, we used SEM and 3D interference microscopy to analyze the microstructure of the pure disordered layer and the patterned layer of the PCL scaffolds. The SEM images revealed a very dense overlap of fibers with no apparent pores (Fig. 1A). Figure 1B displays the 3D morphology of the PCL scaffolds, with different colors representing varying height positions, from red for the top layer to blue for the bottom. The roughness average (Ra) value of the pure disordered layer was 11.37 ± 0.33 μm. The Ra values of P-par, P-rhomb, and P-sq increased to 15.02 ± 0.66 μm, 17.64 ± 0.6 μm, and 17.58 ± 0.79 μm, respectively, indicating that the construction of the patterned layer effectively increased the surface roughness (Fig. 1C). Wettability is a crucial factor in evaluating the influence of biomaterial surfaces on cellular behavior. Hydrophilic surfaces are conducive to cell adhesion, while hydrophobic surfaces can reduce bacterial adhesion [22]. Wettability test revealed that the contact angle of the pure disordered layer was 124.9 ± 2.21°. The construction of the patterned layer enhanced the hydrophilicity of the PCL scaffolds, with contact angles of P-par, P-rhomb, and P-sq decreasing to 114 ± 1.55°, 104.5 ± 1.2°, and 100.77 ± 2.83°, respectively (Fig. 1D and E).

Furthermore, as a soft tissue repair membrane, it is essential to possess favorable mechanical properties. Therefore, we conducted tensile tests on the monolayer disordered PCL (P), P-par, P-rhomb, and P-sq. Figure 1F shows the states of the PCL scaffold before and after the tensile test. The stress–strain curves (Fig. 1G) indicate a rapid increase in stress with strain between 0 and 100% deformation in all groups, followed by a slower increase. Further statistical analysis of the maximum tensile strength and corresponding strain of each group revealed that the P group had the lowest maximum tensile stress at 7.06 ± 0.24 MPa, followed by the P-par group at 9.08 ± 0.43 MPa. Compared to the P group, the P-rhomb and P-sq groups showed an increase in maximum tensile strength of 4.02 and 3.51 MPa, respectively. However, P-rhomb and P-sq showed no statistically significant difference in maximum tensile strength. In terms of strain, the P-rhomb and P-sq groups also exhibited superior performance, with strain increases of 76% and 97% compared to the P group. Overall, the deposition of directional fiber structures effectively enhanced the tensile strength of the PCL scaffold. The cross points between fibers in the P-rhomb and P-sq groups provided additional reinforcement compared to the parallel fiber structure.

### Antibacterial experiments of BIO and PCL

**Fig. 2.**
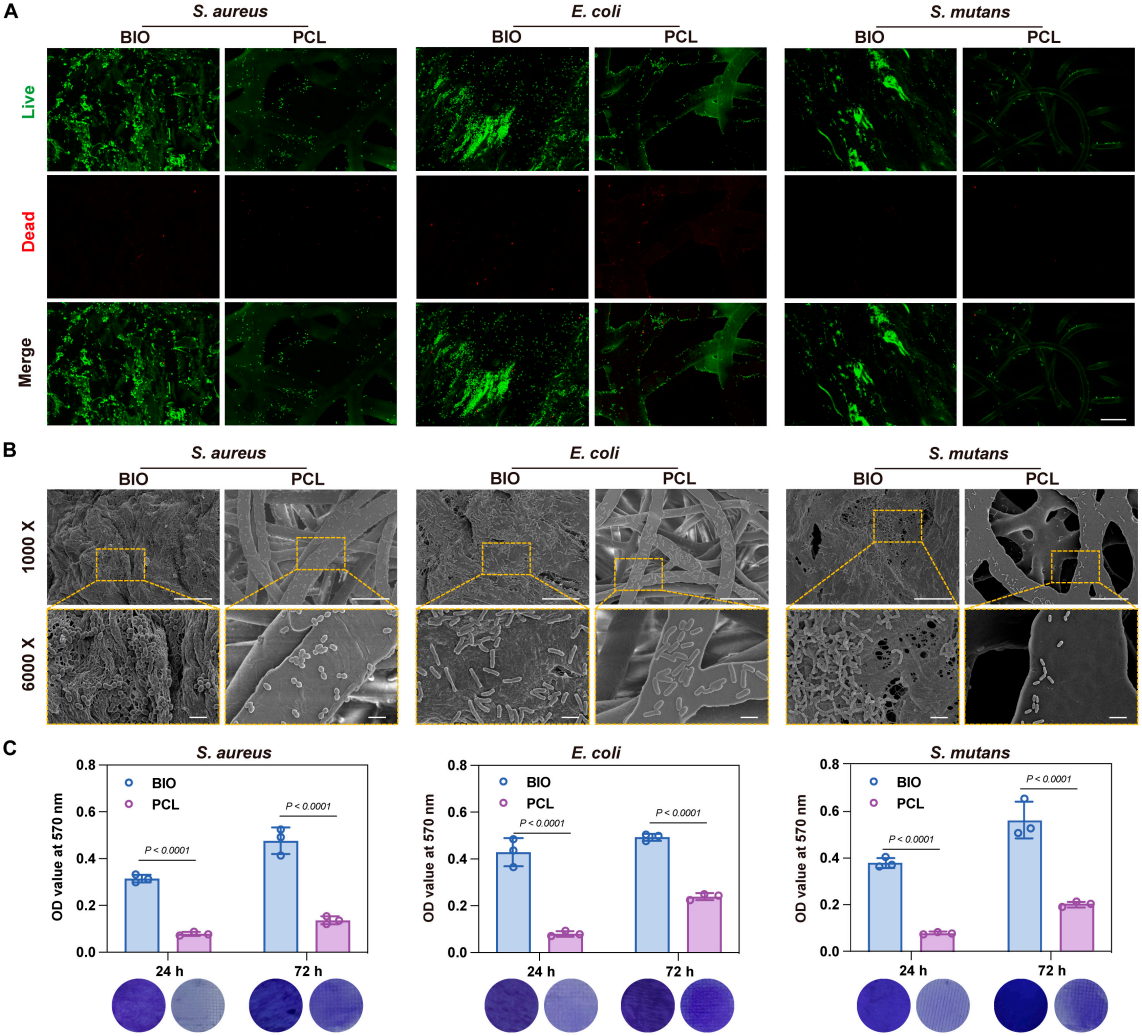
Bacterial adhesion and biofilm formation on the outer surface of commercial collagen membrane and PCL scaffold. (A) Fluorescence images of *S. aureus*, *E. coli*, and *S. mutans*. Scale bar, 20 μm. (B) SEM images of *S. aureus*, *E. coli*, and *S. mutans*. Scale bar, 20 μm. The yellow dashed borders indicate partial magnified images. Scale bar, 5 μm. (C) Representative images and statistical analysis of crystal violet staining. All data are presented as means ± SD; *n* = 3.

Bacterial infections can delay the wound healing process, necessitating soft tissue repair membranes that minimize bacterial adhesion [23]. The composition and surface topography of biomaterials have been extensively investigated, revealing their substainial influence on bacterial adhesion and the formation of biofilms [24,25]. By regulating the surface patterns and roughness, bacteria and biomaterials have a reduced contact area, thereby weakening their initial adhesion [26]. Gram-positive *Staphylococcus aureus* and gram-negative *Escherichia coli* are the common bacteria in infected wounds. *Streptococcus mutans* is the largest genus in the oral flora. Thus, the above 3 bacteria were selected to evaluate the antibacterial function. We used the clinically most commonly used product, Geistlich Bio-Gide (BIO)—a collagen membrane—as a control. Although collagen membranes offer good flexibility, elasticity, and hemostatic properties, they have been shown to serve as a primary substrate for bacterial adhesion and are more conducive to biofilm formation [27]. To evaluate the early bacterial adhesion of BIO and PCL scaffold, *S. aureus*, *E. coli*, and *S. mutans* were inoculated onto the smooth surface of the BIO membrane and the outer layer of the PCL scaffolds for 24 h. Live/dead staining revealed that viable bacteria were observed on both surfaces, while the PCL group exhibited significantly fewer bacteria than the BIO group (Fig. 2A). Additionally, we observed bacterial adhesion on BIO and PCL surfaces by SEM (Fig. 2B). In SEM images, it can be seen that the smooth surface of the BIO membrane is not completely flat but rather uneven, where “grooves” increased the contact area with bacteria, promoting bacterial adhesion. In contrast, the smooth surface of PCL fibers resulted in a reduced contact area, hindering bacterial adhesion. Furthermore, we observed the formation of bacterial biofilms on the BIO and PCL surfaces after 24 and 72 h of inoculation through crystal violet staining (Fig. 2C). The images of the crystal violet staining showed that the bacterial biofilm formed on the BIO surface was more pronounced than that of the PCL group. Therefore, compared to the clinical product BIO, the PCL scaffold demonstrated a superior ability to reduce bacterial adhesion and biofilm formation.

### Biological behavior of HGF-1 on the ordered layer of PCL scaffolds

Good biocompatibility is crucial for medical materials. The cytotoxicity of the PCL scaffold was evaluated using live/dead staining and the CCK-8 assay (Fig. S2A and B). According to the CCK-8 results, compared to the control group, the P-par, P-rhomb, and P-sq groups did not show a significant decrease in cell viability. Additionally, the fluorescence images of live/dead staining did not show an increase in dead cells in P-par, P-rhomb, and P-sq groups. Furthermore, blood clotting, coagulation, and thrombosis are often assessed by hemolysis tests in order to evaluate the adverse effects of biomaterials [28]. As the biomaterials will inevitably come into contact with blood when applied to the human body, it is crucial to assess the blood compatibility of PCL scaffolds. In all groups except the positive control group, the solutions remained clear and transparent (Fig. S2D). In comparison to the negative control group, no significant difference was found in hemolysis between experimental and control groups (Fig. S2C). PCL scaffolds proved to be biocompatible in these studies.

**Fig. 3.**
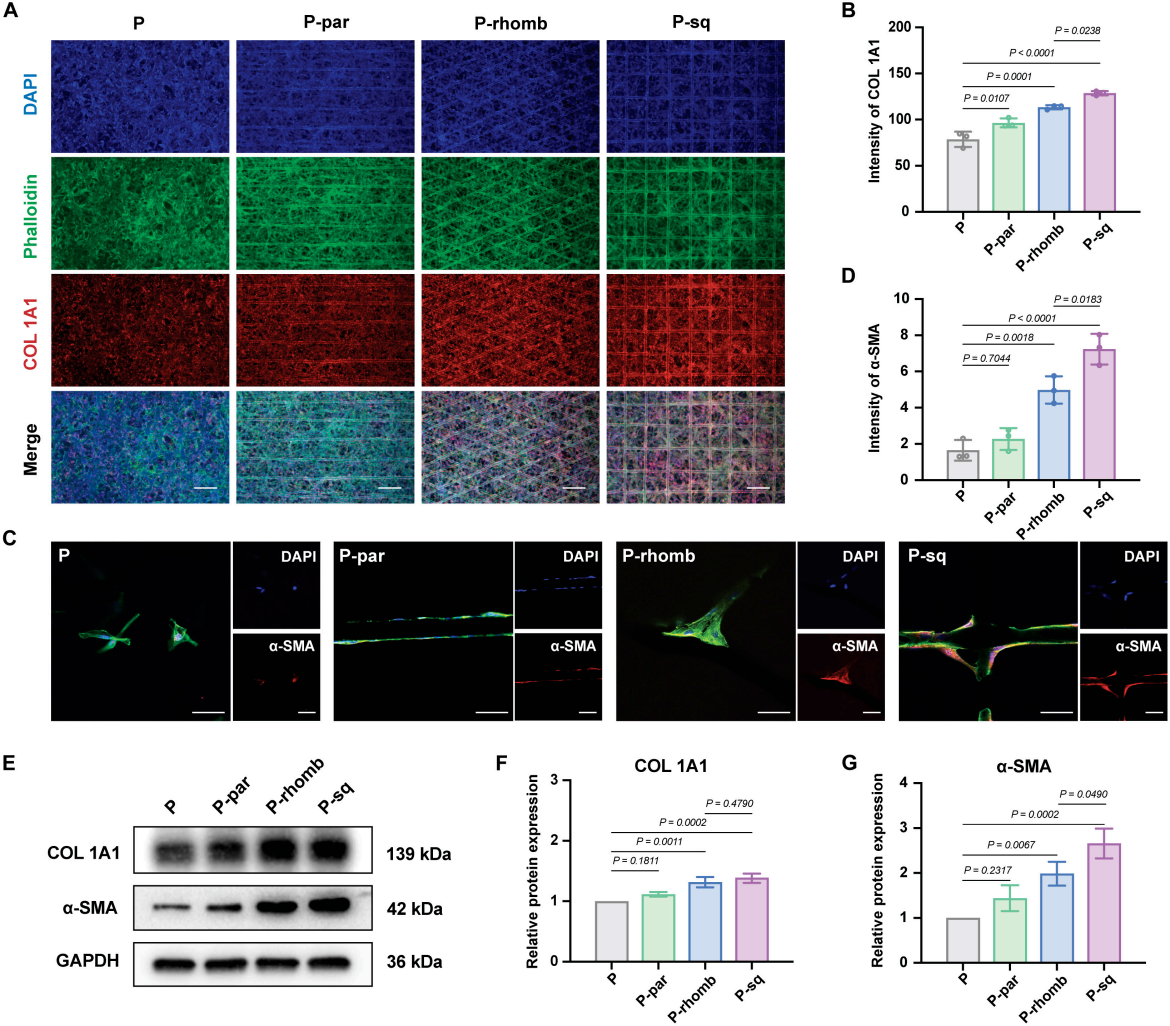
Myofibroblast differentiation of HGF-1 of the PCL scaffolds. (A) Immunofluorescence images of COL 1A1. Scale bar, 200 μm. (B) Semiquantitative fluorescence intensity of COL 1A1. (C) Immunofluorescence images of α-SMA. Scale bar, 50 μm. (D) Semiquantitative fluorescence intensity of α-SMA. (E) Western blot assay of COL 1A1 and α-SMA. (F) Semiquantitative protein expression of COL 1A1. (G) Semiquantitative protein expression of α-SMA. All data are presented as means ± SD; *n* = 3.

Introducing specific fiber patterns can impart controllable biological properties without altering the intrinsic material, playing a crucial role in the functional expression and maintenance of cells on materials [29]. During wound healing, fibroblasts differentiate into a specific transiently expressed phenotype known as “myofibroblasts”, which highly express α-SMA and promote ECM contraction [30]. Myofibroblasts are also an important source of type I collagen. Immunofluorescence results demonstrated that the expression of COL 1A1 was lowest in HGF-1 cells growing on the surface of disordered fibers (P group) and was highest in P-sq (Fig. 3A). Interestingly, the fluorescence intensity of COL 1A1 was higher at the fibrous crossing structures in the P-sq group (Fig. 3B). Furthermore, the expression of α-SMA varied in HGF-1 cells grown on the fibers with different crossing angles. The square pattern with 90° fiber crossing structures can significantly promote the differentiation of gingival fibroblasts into myofibroblasts (Fig. 3C and D). Furthermore, Western blot results also confirmed that COL 1A1 and α-SMA protein expression in the P-sq and P-rhomb groups was significantly higher than that in the P and P-par groups (Fig. 3E to G), which was consistent with the result of immunofluorescence staining. Overall, the P-sq group had the highest expression of COL 1A1 and α-SMA. This may be related to the YAP protein, which is associated with mechanical stress. In a previous study on how the scaffold structure with different fiber angles affects cell behavior, it was found that human skeletal stem cells seeded on the 90° fibrous scaffold had the highest expression of YAP protein [31]. Immunofluorescence staining was used to investigate the expression of YAP protein of HGF-1 cells on the surface of different fibrous structures. As we speculated, the expression of YAP protein in HGF-1 cells was most pronounced in the P-sq group (Fig. S3). YAP protein acts as a tissue mechanosensor and can be activated by mechanical stress, promoting and maintaining fibrosis. In the development of fibrosis, YAP promotes fibroblast differentiation into myofibroblasts and up-regulates α-SMA expression [32,33]. Therefore, we believe that YAP is activated in HGF-1 on 90° fibrous scaffold, which in turn up-regulates the expression of COL 1A1 and α-SMA, and ultimately promotes HGF-1 differentiation into myofibroblasts

**Fig. 4.**
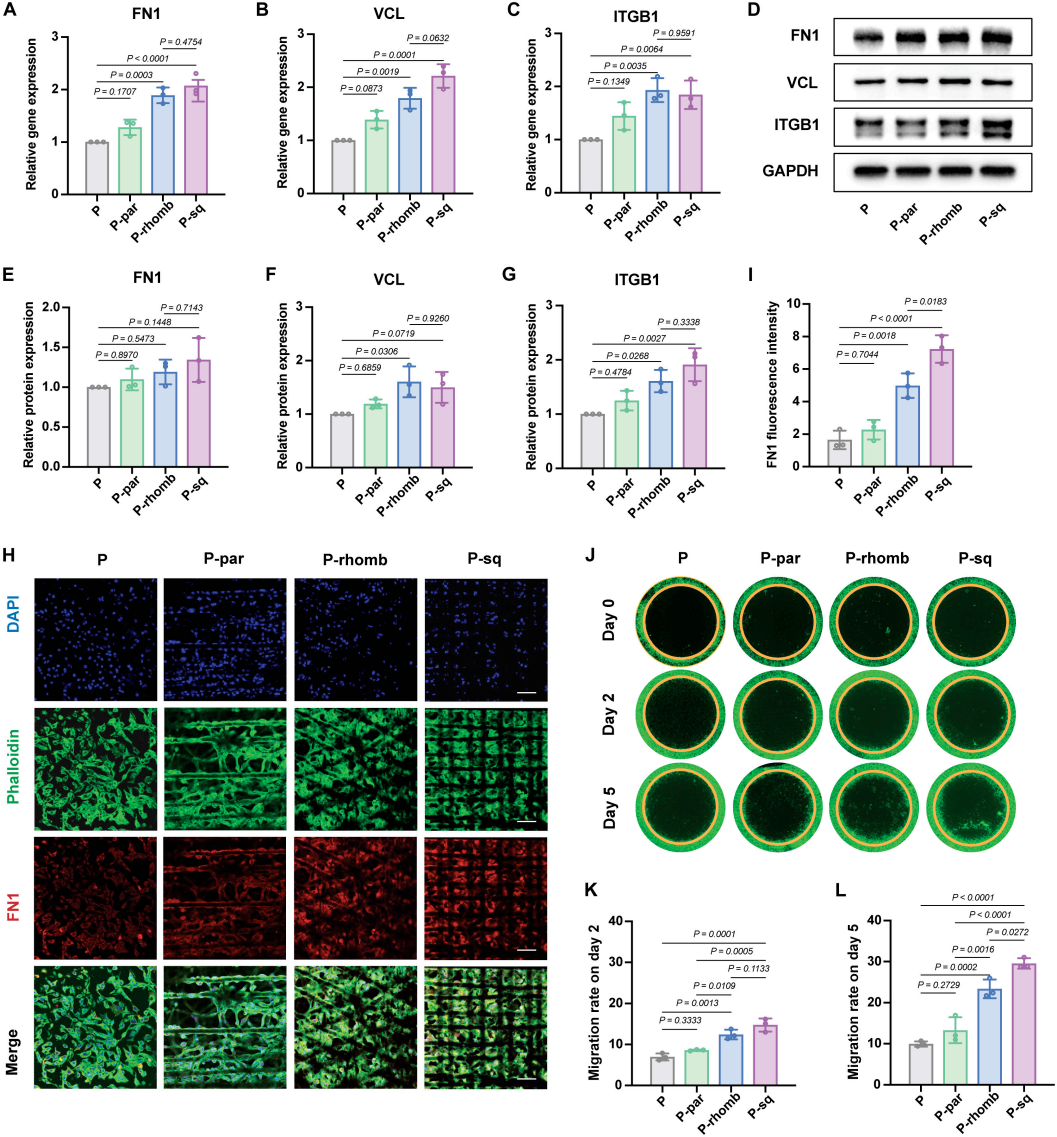
Adhesion and migration of HGF-1 of the PCL scaffolds. (A) Gene expression of FN1. (B) Gene expression of ITGB1. (C) Gene expression of VCL. (D) Western blot assay of FN1, ITGB1, and VCL. (E) Semiquantitative protein expression of FN1. (F) Semiquantitative protein expression of ITGB1. (G) Semiquantitative protein expression of VCL. (H) Immunofluorescence images of FN1. Scale bar, 250 μm. (I) Semiquantitative fluorescence intensity of FN1. (J) Migration of HGF-1 on PCL scaffolds. (K) Migration rate of HGF-1 on day 2. (L) Migration rate of HGF-1 on day 5. All data are presented as means ± SD; *n* = 3.

Generally, a rough surface structure can increase the contact area between the material and cells, promoting cell adhesion, differentiation, and proliferation [34]. Common adhesion-related proteins include ITGB1, VCL, and FN1. FN1 is a structural and biochemical component of the ECM, with multiple interaction sites containing cell adhesion molecules (including integrins and heparan sulfate) [35]. ITGB1 plays an important role in diffusion, migration, and signal transduction by providing a transmembrane connection between the intracellular and extracellular matrix [36]. As an integral part of adhesion plaques, VCL links integrin adhesion molecules to actin cytoskeletons [37]. After culturing HGF-1 cells on the scaffolds in P, P-par, P-rhomb, and P-sq scaffolds for 48 h, reverse transcription polymerase chain reaction (RT-PCR), Western blot assay, and immunofluorescence staining were performed to study the adhesion of HGF-1 on PCL scaffolds of different patterns. RT-PCR results showed significantly higher expression of FN1, ITGB1, and VCL mRNA expression in the P-rhomb and P-sq groups compared to the P group (Fig. 4A to C). In addition, Western blot results confirmed the elevated expression of FN1, ITGB1, and VCL (Fig. 4D to G), which was largely consistent with the RT-PCR results. The gene and protein expression levels of VCL and ITGB1 were not consistent between the P-sq and P-rhomb groups. Nevertheless, no statistically significant difference was observed between the 2 groups. It is evident that both the P-sq and P-rhomb groups exhibited significantly higher expression levels than did the P group. As shown in Fig. 4I, HGF-1 cultivated on P-sq exhibited the strongest fluorescence for FN1 (red), followed by P-rhomb and P-par. The quantitative statistics revealed that the P group expressed much less FN1 than P-par, P-rhomb, and P-sq (Fig. 4H). These results indicate that the P-rhomb and P-sq surfaces are more conducive to early cell adhesion than the P and P-par surfaces, which may be attributed to the better hydrophilicity and rougher surface. These genes are related to the formation and regeneration of soft tissue. The increased expression of these genes suggests that the ability of HGF-1 to form soft tissue and promote wound healing is enhanced.

Furthermore, we investigated the migration behavior of HGF-1 on PCL scaffolds with different fiber patterns (Fig. 4J to L). From days 0 to 5, HGF-1 on the P-rhomb and P-sq surfaces exhibited higher migration rates than the random and parallel fiber surfaces. Statistical analysis revealed that on the fifth day, the cell migration rates of the P-sq and P-rhomb groups reached 29.56% and 23.37%, which were 2.97 and 2.35 times higher than that of the P group (9.94%), respectively. Although there were no significant differences in cell migration rates between the P and P-par groups at 2 and 5 d, the P-par group demonstrated migration rates of 8.6% and 13.3%, respectively, which were higher than those of the P group. The difference in roughness and wettability of different fiber patterns is one of the factors that affect the migration of HGF-1. Furthermore, the fiber pattern of each group affected the differentiation of myofibroblasts, which in turn altered cell adhesion and migration. According to these findings, the cross-fiber pattern encourages cell migration and promotes wound healing.

### In vivo wound healing performance

**Fig. 5.**
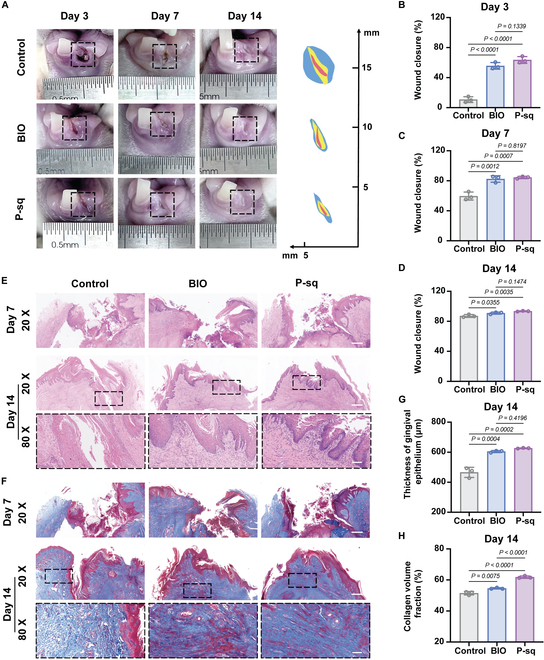
In vivo evaluation of BIO and P-sq scaffold treatment in oral full-thickness mucosal wound. (A) Mucosa restoration images. Wound closure rate on (B) day 3, (C) day 7, and (D) day 14. (E) Representative H&E staining images. Scale bar, 500 μm. The black dashed borders indicate partial magnified images. Scale bar, 100 μm. (F) Representative Masson staining images. Scale bar, 500 μm. The black dashed borders indicate partial magnified images. Scale bar, 100 μm. (G) Thickness of the new epithelium on day 14. (H) Collage volume fraction on day 14. All data are presented as means ± SD; *n* = 3.

Given the mechanical properties of the PCL scaffold and its ability to induce HGF-1 migration and exert biological functionality, P-sq was selected for subsequent animal experiments. A gingival defect model was created by extracting the lower left incisor in New Zealand rabbits, and the ability of P-sq to repair oral full-thickness mucosal defects was investigated. We used the untreated defect group as a negative control (the control group) and BIO implantation as a positive control (the BIO group). Figure 5A presents the overall images of oral mucosal recovery on days 3, 7, and 14 after surgery in the control, BIO, and P-sq groups. An examination of the mucosal recovery at 3 d after surgery revealed that the control group did not recover as well as the BIO and P-sq groups. Wound area was measured in rabbits from each group on days 3, 7, and 14 after recovery using ImageJ software. Quantitative analysis revealed that the wound closure rate in the P-sq group on day 3 after surgery was 63.56% ± 4.47%, which was 5.9 times higher than the control group (10.72% ± 3.83%) and even slightly better than the BIO group (55.63% ± 4.41%) (Fig. 5B). The wound healing rates at 7 d after surgery for the control, BIO, and P-sq groups were 59.59% ± 5.38%, 82.36% ± 4.34%, and 84.40% ± 1.55%, respectively (Fig. 5C). By the 14th day after surgery (Fig. 5D), the mucosal wounds had healed significantly in all groups, with wound healing rates exceeding 90% in the BIO and P-sq groups, with P-sq showing the best performance and the control group showing the worst (87.18% ± 1.88%). These results suggest that both BIO and P-sq are effective in the early stage of mucosal wound repair, with P-sq exhibiting particularly impressive performance.

### Histology evaluation

Hematoxylin and eosin (H&E) staining was used to investigate the recovery of the mucosa on the regenerated tissues. Figure 5E shows the histological section and local magnification of the mucosal injury. On day 7 after surgery, the control group exhibited only a small amount of regenerated epithelial cells in the mucosal defect site, with no apparent epithelial stratification, and intense inflammatory cells in the submucosa. The BIO group had more epithelial cells at the defect site than the control group, but no continuous epithelial structure was formed. In the P-sq group, the epithelium was stratified, with a thick and continuous epithelial structure, possibly due to the directional fiber structure of P-sq accelerating the growth of epithelial cells around the wound. On day 14 after surgery, the control group showed continuous epithelial tissue but lacked apparent layering and peak structures. The BIO group had visible epithelial pegs, but their morphology was poor. In contrast, the regenerated mucosa in the P-sq group exhibited prominent epithelial pegs with a normal morphology and uniform basal layer thickness. Quantitative analysis revealed that at 14th day after surgery, the gingival epithelium regenerated in the P-sq group was the thickest at 626.7 ± 2.28 μm, slightly lower in the BIO group (603 ± 5.7 μm), and the thinnest in the control group at 465.62 ± 33.84 μm (Fig. 5G).

During the wound healing process, fibroblasts begin synthesizing and secreting a large amount of collagen fibers and matrix components from the 5th to 6th days of repair, together with the formation of new capillaries. They gradually form granulation tissue and fill the wound defect site, creating conditions for the coverage of epithelial cells [38]. To investigate collagen formation at the wound sites, we employed Masson’s trichrome staining (Fig. 5F). Consistent with the H&E results, the wound healing situation covered with the BIO and P-sq scaffolds was superior to that of the control group. The regenerated mucosa in the P-sq group on day 7 after surgery exhibited a favorable morphology, with clear layering and a continuous epithelial structure. On day 14 after surgery, the regenerated mucosa in the control group showed poor recovery, with disordered arrangement of collagen fibers in the submucosa, while the P-sq group had regularly deposited collagen fibers underneath the epithelium. Moreover, collagen volume calculations indicated that the P-sq group formed more collagen than the BIO group, while the control group formed the least (Fig. 5H). It appears that the P-sq scaffold induces mucosal regeneration more effectively in the rabbit oral mucosal defect model.

**Fig. 6.**
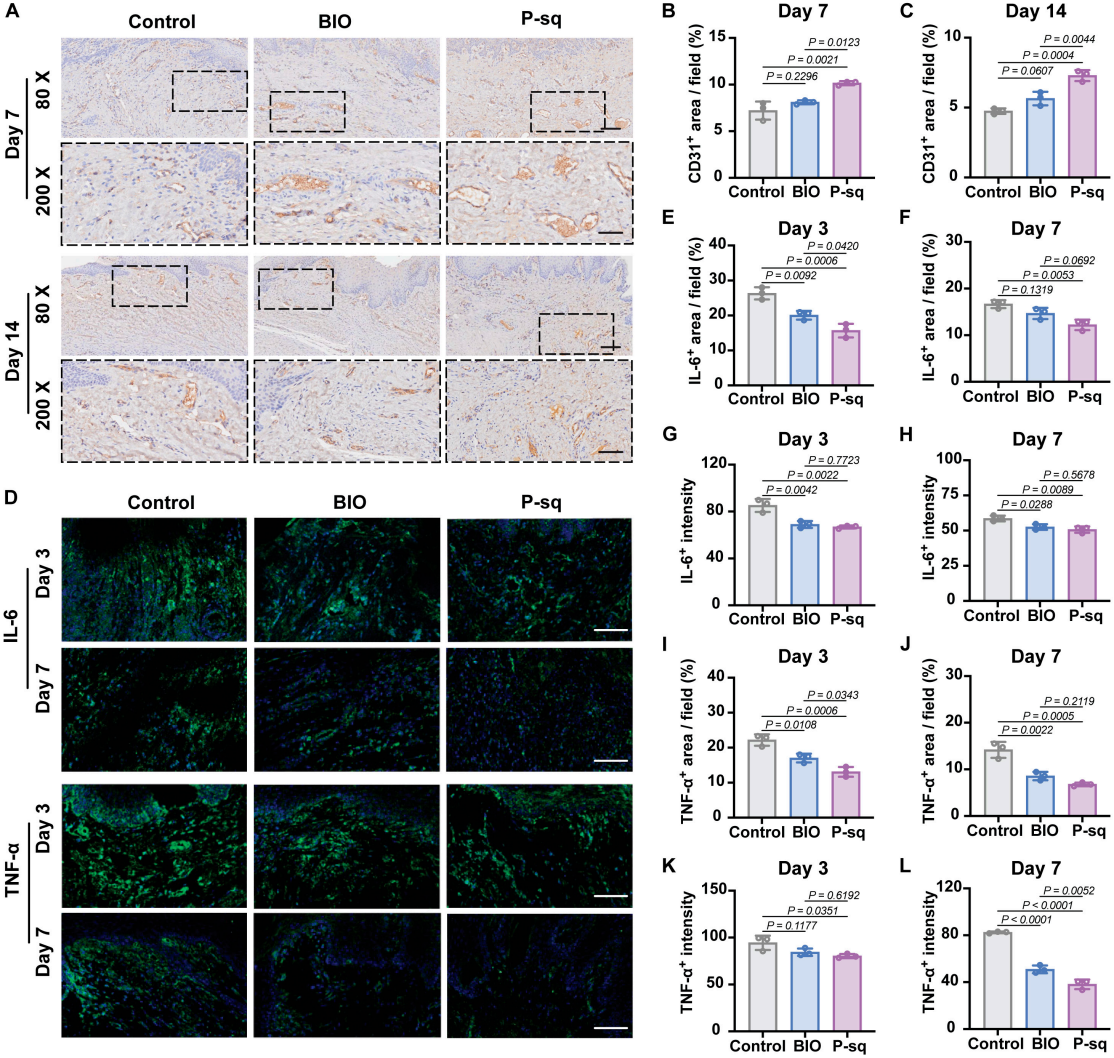
The histological analysis of BIO and P-sq scaffold treatment in oral full-thickness mucosal wound. (A) Immunohistochemical staining images of CD31. Scale bar, 100 μm. The black dashed borders indicate partial magnified images. Scale bar, 50 μm. Quantitative analysis of CD31^+^ area on (B) day 7 and (C) day 14. (D) Immunofluorescence histochemical staining images of IL-6 and TNF-α. Scale bar, 100 μm. Quantitative analysis of IL-6^+^ area on (E) day 3 and (F) day 7. Quantitative analysis of IL-6^+^ intensity on (G) day 3 and (H) day 7. Quantitative analysis of TNF-α^+^ area on (I) day 3 and (J) day 7. Quantitative analysis of TNF-α^+^ intensity on (K) day 3 and (L) day 7. All data are presented as means ± SD; *n* = 3.

We evaluated the level of vascularization in the defect areas of each group using IHC labeling of CD31^+^ cells, as vascular generation is a critical component of tissue regeneration [39]. The brownish yellow labeling in Fig. 6A represents the CD31^+^ signals, indicating the formation of vessels. Both the BIO and P-sq groups exhibited significantly enlarged newly formed blood vessels 7 d after surgery. As vascular generation matured, the number of CD31^+^ vessels decreased, and the vessel diameter was reduced by 14th day after surgery. Compared with control and BIO groups, the P-sq group had a significantly higher amount of CD31^+^ cells infiltrating the vascular system (Fig. 6B and C). Biomaterial surfaces with directional morphology have been reported to enhance the expression of genes and proteins related to vascular development [40]. The above results confirm that P-sq can promote vascular reconstruction during the wound healing process.

To further explore the inflammatory response during the wound healing process in each group, we performed immunofluorescent staining for tumor necrosis factor-α (TNF-α) and interleukin-6 (IL-6) (Fig. 6D). According to the quantitative results, the control group exhibited the highest levels of IL-6- and TNF-α-positive areas, while the IL-6- and TNF-α-positive areas in the BIO and P-sq groups were notably reduced (Fig. 6E, F, I, and J). Furthermore, the fluorescence intensity of IL-6 and TNF-α in the BIO and P-sq groups was also significantly lower than that in the control group (Fig. 6G, H, K, and L), although there was no significant difference between P-sq and BIO. These findings suggest that the BIO and P-sq groups had significantly reduced levels of IL-6 and TNF-α expression compared to the control group. PCL scaffolds offer a barrier effect that inhibits the adhesion of bacteria and reduces the risk of infection, which may explain the lower level of inflammatory cytokines. In conclusion, P-sq can promote mucosal regeneration and wound healing by inhibiting bacterial adhesion, reducing wound infection, suppressing inflammation, and promoting vascular generation.

## Conclusion

In summary, this study prepared an integrated dual-layer heterogeneous PCL scaffold using the MEW technique, consisting of a disordered outer layer and a patterned inner layer. The structural heterogeneity of the PCL scaffolds enables them to simultaneously achieve the dual functions of inhibiting bacterial biofilm formation and promoting cell adhesion, migration, and differentiation. The integrated scaffold can be easily prepared by adjusting the printing parameters, eliminating the need for complex cross-linking systems or components. In a rabbit model with full-thickness gingival defects, P-sq was found to decrease inflammation and promote vascular regeneration, ultimately accelerating epithelial regeneration and wound healing. Therefore, the easy customization of the integrated dual-layer heterogeneous PCL scaffold, fabricated using the MEW technique, acts as a barrier to reduce inflammation and bacterial invasion, and simultaneously, fiber-pattern structures induce rapid oral mucosal wound healing. This novel strategy offers an effective approach for the rapid healing and regeneration of full-thickness oral mucosal defects, demonstrating potential for clinical application and large-scale production.

## Materials and Methods

### Fabrication of the dual-layer PCL scaffolds

A MEW device (BP6601-X, SuZhou Intelligent Manufacturing Research Institute, SuZhou, China) was used to deposit the dual-layer PCL scaffold. The outer layer of the PCL scaffold was printed in a disordered manner with the following settings: nozzle temperature, 90 °C; air pressure, 1.5 kPa; voltage, 12 kV; distance between the collector and the nozzle, 10 mm; and collector speed, 1,000 mm/min. The fiber spacing of the disordered layer was 15 μm. Three directional fiber structures were then printed on the disordered layer: P-par, P-rhomb, and P-sq. The fiber spacing of the ordered layer was 200 μm. Under the given conditions of a nozzle temperature of 90 °C, air pressure of 10 kPa, voltage of 4.5 kV, distance of 2.5 mm between the collector and the nozzle, and a collector speed of 800 mm/min, the ordered layers with varying fiber cross angles were printed for 3 layers.

### Characterization of the dual-layer PCL scaffolds

The surface morphology of the dual-layer PCL scaffolds was observed by SEM (GEMINI 300, ZEISS, Germany). First, the samples were coated with a layer of gold-palladium using a sputtering device. Next, the 3D topography and roughness were evaluated by 3D interference microscopy (NT9100, Veeco, USA). Subsequently, the water contact angles were measured using an OCA15p instrument (DataPhysics, Germany) by the sessile drop method. UTM2102 (Shenzhen Sun Technology Co. Ltd.) was used to measure the tensile mechanical characteristics of pure disordered PCL (P), P-par, P-rhomb, and P-sq at a tensile rate of 2 mm·min^−1^.

### Antibacterial experiments

#### Bacteria live-dead staining

To evaluate bacterial viability on different materials (Geistlich Bio-Gide and PCL), *E. coli*, *S. aureus*, and *S. mutans* were utilized. Briefly, Geistlich Bio-Gide and PCL were immersed in the bacterial suspensions [1 × 10^8^ colony-forming units (CFU)/ml] and incubated for 24 h at 37 °C. Bacteria were then stained with LIVE/DEAD BacLight Bacterial Viability Kit (Thermo Fisher Scientific, USA) according to the manufacturer’s instructions. Finally, stained bacteria were observed and photographed using a confocal laser scanning microscope (CLSM; Leica Microsystems, Germany).

#### Bacterial adhesion assay

Geistlich Bio-Gide and PCL were submerged in the bacterial solution (1 × 10^8^ CFU/ml) and gently shaken for 24 h at 37 °C to assess the bacterial adhesion ability. After washing 3 times using phosphate-buffered saline (PBS) to get rid of unattached bacteria, the samples were fixed with 2.5% glutaraldehyde. After that, the sample was dehydrated with a series of ethanol solutions (30%, 50%, 70%, 80%, 90%, 95%, and 100%) for 15 min. Lastly, bacterial adhesion was observed using the SEM.

#### Bacteria crystal violet staining

In order to observe the formation of bacterial biofilms, bacterial suspensions (1 × 10^8^ CFU/ml) were added to Geistlich Bio-Gide and PCL, and cultured for 24 and 72 h at 37 °C. Following a 15-min room temperature fixation with 95% methanol, the samples were stained using a 0.5% crystal violet staining solution for 15 min. After the digital camera photographs were taken, the samples were decolorized with 95% ethanol, and a microplate reader was used to measure the eluent’s absorbance values at 570 nm.

### Live-dead assay

HGF-1 cells were purchased from Bluefbio Biology Technology Development Co. Ltd. (Shanghai, China) and cultured in Dulbecco’s modified Eagle’s medium (Gibco, USA) supplemented with 10% fetal bovine serum (Gibco, USA) and 1% penicillin-streptomycin solution (Sigma, USA). In the live-dead assay, the samples were cocultured with HGF-1 cells for 1, 3, and 5 d using Transwell chambers. HGF-1 cells cocultured without samples were used as control group. Subsequently, calcein-AM (Bestbio, China) and propidium iodide were used for live-dead staining. Finally, fluorescence microscopy (Invitrogen EVOS M5000, USA) was utilized to observe and capture images of the stained cells.

### CCK-8 assay

A CCK-8 assay was performed to assess the cytotoxicity of the PCL scaffolds following the manufacturers’ instructions. Briefly, HGF-1 were seeded in the 24-well tissue culture plate and the samples were added in the upper chamber of Transwell plates. After coculturing with cells over 1, 3, and 5 d, CCK-8 solution (Beyotime, China) was added into the culture plate and cultured for 2 h at 37 °C. A microplate reader (Perkin-Elmer, USA) was used to measure the optical density (OD) at 450 nm.

### Hemolysis assay

The blood compatibility assessment of the PCL scaffolds was performed utilizing an in vitro hemolysis assay. Fresh anticoagulated whole rabbit blood (Shanghai Yuanye Bio-Technology Co. Ltd.) was appropriately diluted with 0.9% normal saline. Then, the samples were added to diluted blood and incubated for 30 min at 37 °C. The samples from different groups were supplemented with normal saline employed as the negative control and ultrapure water (UPW) as the positive control.

 Afterward, 200 μl of diluted blood was added to each centrifuge tube andincubated for 1 h at 37 °C. The tubes were then centrifuged at 800*g* for 5 min. The OD values of the supernatant were measured at 540 nm, and the hemolysis rate was calculated using the following formula:

$$\text{Hemolysis rate}\;\left(\%\right)=\frac{\text{ODsample}-\text{ODnegative}}{\text{ODpositive}-\text{ODnegative}}\times 100$$

Here, ODsample indicates the OD value of the liquid supernatant from distinct groups, ODpositive stands for the OD value of the UPW positive control, and ODnegative implies the OD value of 0.9% normal saline.

### Immunofluorescence staining

HGF-1 cells were enzymatically digested using 0.25% trypsin and subsequently seeded onto PCL scaffolds. After 24 h of incubation, the samples were fixed, permeabilized, and incubated with fibronectin1 (FN1; Huabio, China), collagen type I (COL 1A1; Proteintech, USA), α-SMA (Proteintech, USA), and YAP1 (Proteintech, USA) antibodies overnight at 4 °C. The cells were then incubated with the secondary antibody for 1 h at room temperature. The cells were then treated with 70 nM fluorescence phalloidin (Cytoskeleton Inc., USA) for 30 min and 5 μg/ml 4′,6-diamidino-2-phenylindole (DAPI) (Solarbio, Beijing, China) for 5 min. The CLSM (Leica Microsystems, Germany) was used to capture the images, and fluorescent intensity quantification was performed with ImageJ software.

Cell migration was observed as the following procedure: First, the HGF-1 cells were seeded onto the inner layer of the scaffolds, which were covered by titanium rods (Φ = 5 mm). Rods were removed after 24 h of incubation to guide cell migration, and the covered areas were calculated at 2 and 5 d. Cells were stained with phalloidin after fixation and permeabilization.

### Reverse transcription polymerase chain reaction

After culturing HGF-1 on PCL scaffolds for 48 h, the TRIZOL reagent (Invitrogen, USA) was used to extract total RNA and the PrimeScript RT reagent kit (Takara Bio, Japan) was used to reverse transcribe RNA to cDNA. Commercially synthesized gene primers (Sangon Biotech Co. Ltd., China) of FN1, integrin β-1 (ITGB1), and vinculin (VCL) were used, and the specific primer sets were detailed in Table S1. A combination of BioEasy SYBR Green I, cDNA templates, and primers were used for quantitative real-time PCR analysis. The mRNA levels were normalized to glyceraldehyde-3-phosphate dehydrogenase (GAPDH) expression using the comparative Ct (2^−ΔΔCt^) method.

### Western blot

After culturing HGF-1 on PCL scaffolds for 48 h, the total protein was extracted by radioimmunoprecipitation assay (RIPA) lysis buffer (Beyotime, China) and separated by sodium dodecyl sulfate polyacrylamide gel electrophoresis (SDS-PAGE). Then, protein was transferred onto a polyvinylidene difluoride membrane (Millipore, MA, USA), and the membrane were blocked with 5% skim milk at room temperature for 2 h. Membranes were incubated overnight at 4 °C with primary antibodies including FN1, ITGB1 (Proteintech, USA), VCL (Proteintech, USA), COL 1A1, and α-SMA, followed by secondary antibodies for 1 h at room temperature. Protein bands were visualized by a chemiluminescence gel imaging system (Bio-Rad, USA), and quantitative analysis was performed with the ImageJ software.

### Animal experiments

All animal experimentation procedures were performed according to the guidelines approved by the Medical Laboratory Animal Centre of Zhejiang Province (Hangzhou, China). The laboratory animals were cared for and used following the Chinese National Institutes of Health Guidelines for the Care and Use of Laboratory Animals. Study approval was obtained from the Medical Ethics Committee of Zhejiang University (approval no. ZJU20230184). For the purposes of this investigation, healthy male New Zealand white rabbits (aged 3 months, weighing between 2 and 2.5 kg) were used as the model for the animal experiments in this study. Thirty rabbits were randomly divided into 3 groups: control group, BIO group, and P-sq group.

#### Establishing the animal models

After a 2-week adaptation period, the rabbits underwent anesthesia, and their left incisors were removed. The untreated injuries formed the control group, while the wounds sutured with Geistlich Bio-Gide comprised the BIO group. The P-sq group was treated with a P-sq scaffold to protect the injury. Gingival healing photographs were captured at the 3rd, 7th, and 14th postoperative days using a digital camera. The rabbits were euthanized on days 3, 7, and 14 after surgery, and the tissues collected were used for histological examination.

#### Histological analysis

The collected rabbit mandibles were fixed in 4% paraformaldehyde, decalcified using 10% ethylenediaminetetraacetic acid, and embedded in paraffin. The tissues were sectioned into 5-μm portions to allow for H&E and Masson’s trichrome staining, which allowed the assessment of the healing process of the rabbit gingiva. In addition, the tissues were also processed with IHC analysis (CD31) and immunofluorescence staining analysis (IL-6 and TNF-α) to assess the regeneration of epithelial tissues. The pictures were acquired using the Olympus digital slice scanner (VS200, Japan) and subsequently analyzed quantitatively with ImageJ.

### Statistical analysis

For robustness and reliability, experiments were performed at least 3 times. Data are presented as mean ± standard deviation (SD). Statistical analysis was performed using GraphPad Prism 8 software (GraphPad Prism Software, USA) with 1- or 2-way analysis of variance (ANOVA). Values of *P* < 0.05 were considered to be statistically significant.
