# Supplementary material for: An Integrated Dual-Layer Heterogeneous Polycaprolactone Scaffold Promotes Oral Mucosal Wound Healing through Inhibiting Bacterial Adhesion and Mediating HGF-1 Behavior
Source: Research (Wash D C). 2024 Oct 24;7:0499. doi: 10.34133/research.0499 (PMC11651385; doi:10.34133/research.0499)
Supplement: Supplementary 1 — Figs. S1 to S3 Table S1 [file research.0499.f1.zip › Supplementary Information.docx]

**Supplementary Materials**

**Table S1**. RT-PCR genes and primer sequences.

| **Gene** | **Primer** | **Sequence (5’ - 3’)** |
| --- | --- | --- |
| FN1 | Forward | AGAGGCATAAGGTTCGGGAAGAGG |
|  | Reverse | CGAGTCATCCGTAGGTTGGTTCAAG |
| ITGB1 | Forward | TGGGCTTTACGGAGGAAGTAGAGG |
|  | Reverse | GACACTTGGGACTTTCAGGGATGC |
| VCL | Forward | GCTCTGCTGATGGCTGAGATGTC |
|  | Reverse | GGCGATGTCCTTGGCACACTG |
| GAPDH | Forward | GCTCTCTGCTCCTCCTGTTC |
|  | Reverse | GACTCCGACCTTCACCTTCC |


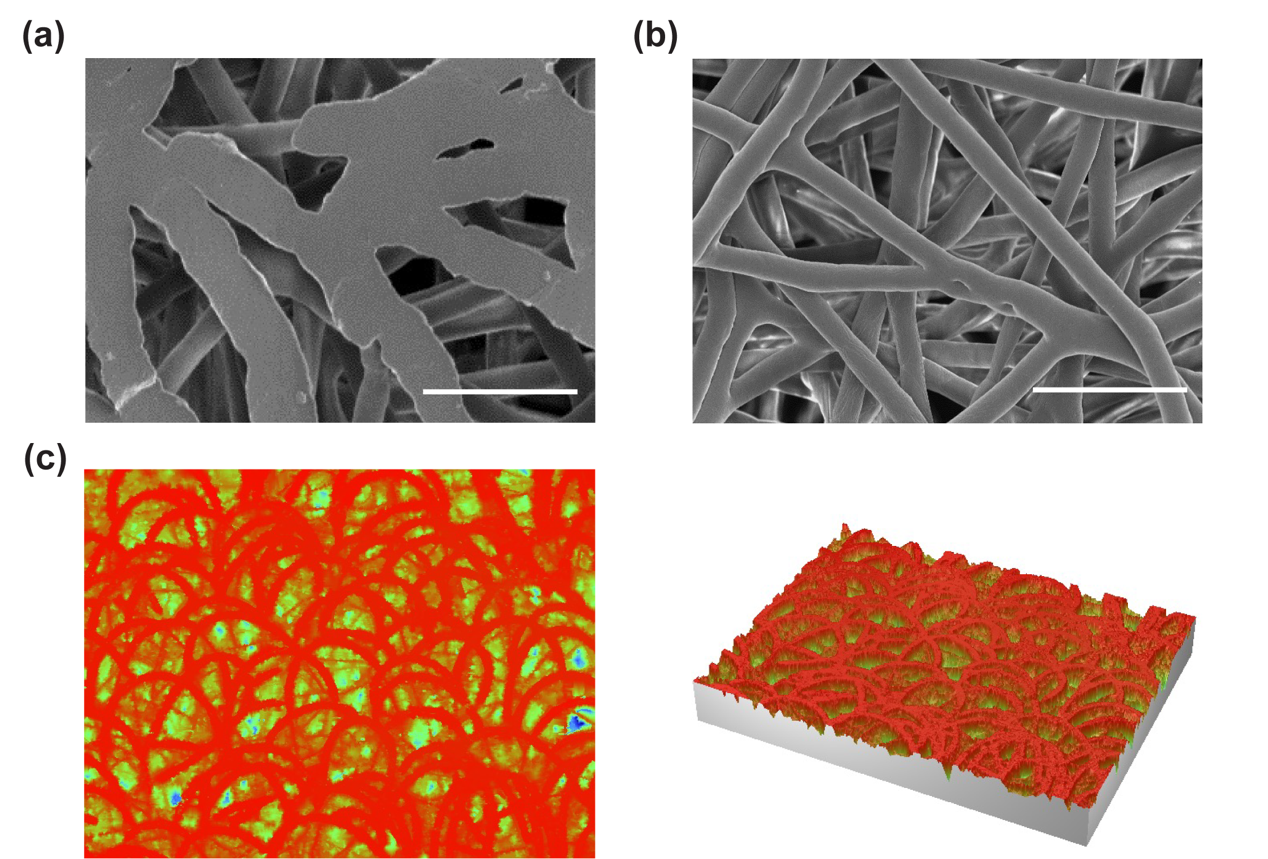


**Fig. S1.** SEM images of the outer (a) and inner (b) surface of the disordered layer, scale bar = 25 μm. (c) Microprofilometer images of the outer surface of the disordered layer.


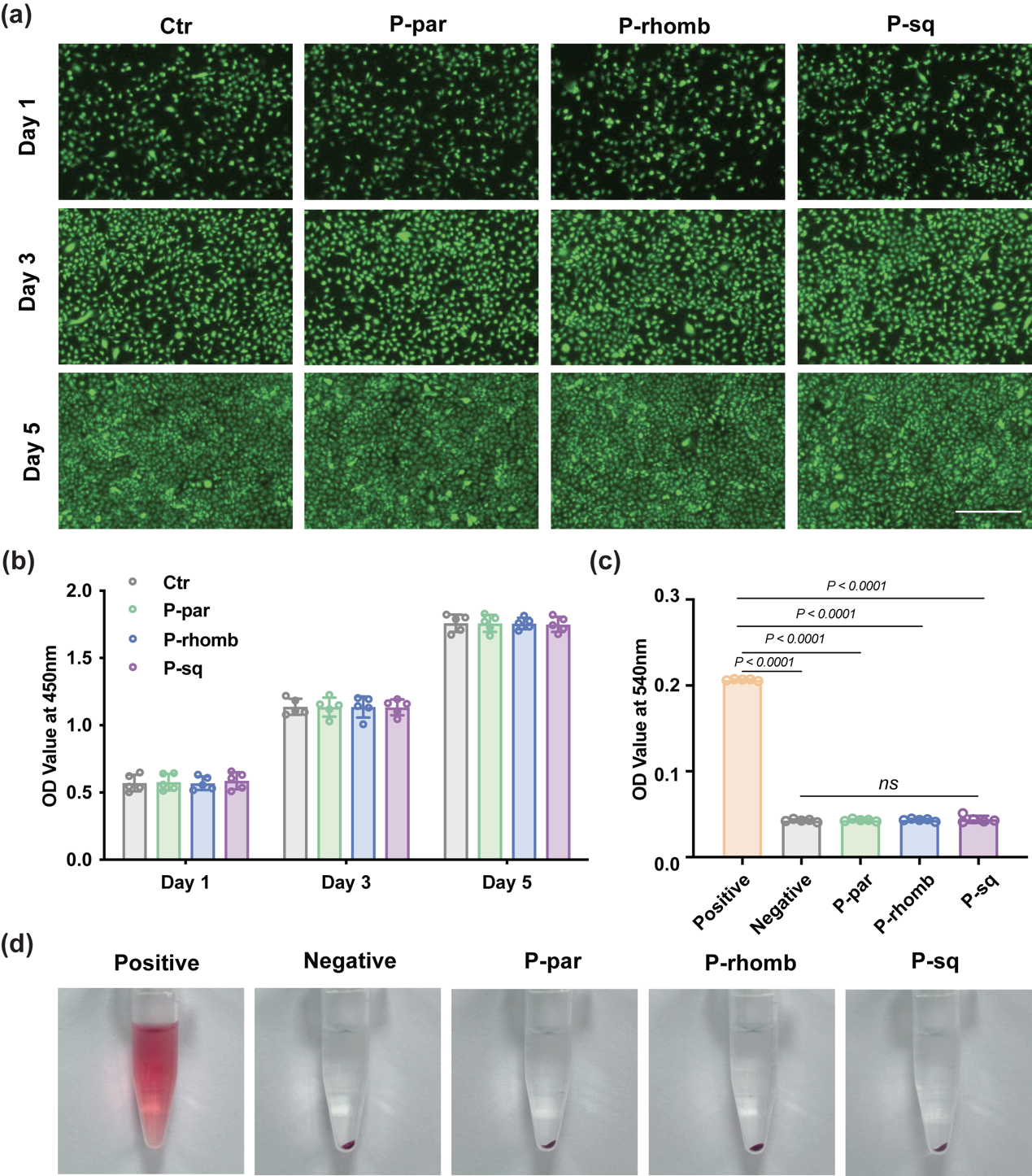


**Fig. S2.** Biocompatibility of PCL scaffolds. (a) The fluorescence images of live-dead staining, scale bar = 250 μm. (b) CCK-8 assay. (c) Hemolysis assay. (d) Representative images of Hemolysis test. (All data are presented as means ± SD, n=5).


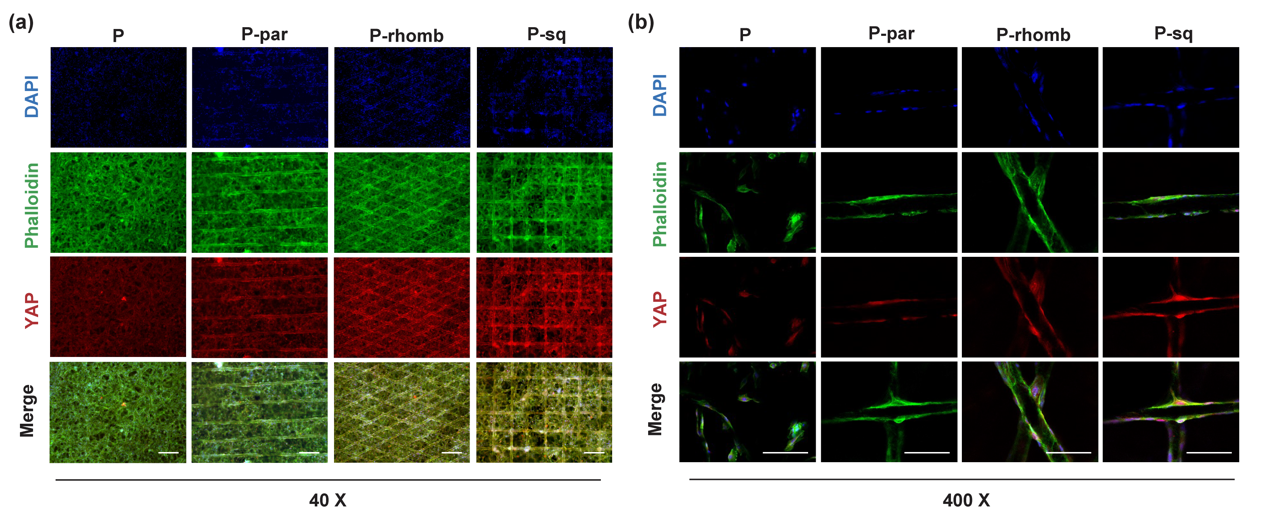


**Fig. S3.** Immunofluorescence images of YAP protein expression at 40 × magnification (a) and at 400 × magnification (b).
