# Supplementary figures and images for: An Integrated Dual-Layer Heterogeneous Polycaprolactone Scaffold Promotes Oral Mucosal Wound Healing through Inhibiting Bacterial Adhesion and Mediating HGF-1 Behavior
Source: Research (Wash D C). 2024 Oct 24;7:0499. doi: 10.34133/research.0499 (PMC11651385; doi:10.34133/research.0499)

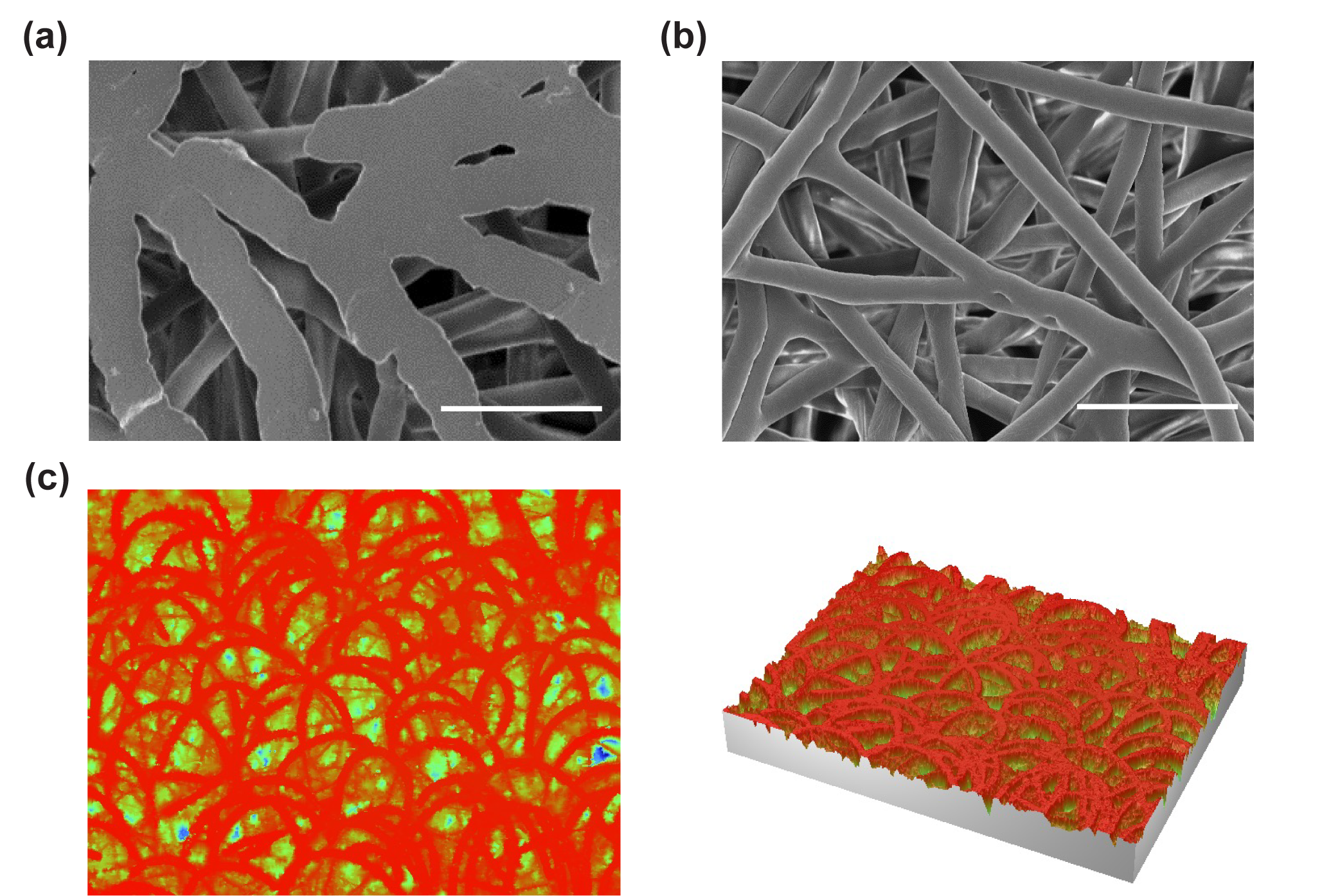

Supplement: Supplementary 1 — Figs. S1 to S3 Table S1 [file research.0499.f1.zip › Figure S1.tif]

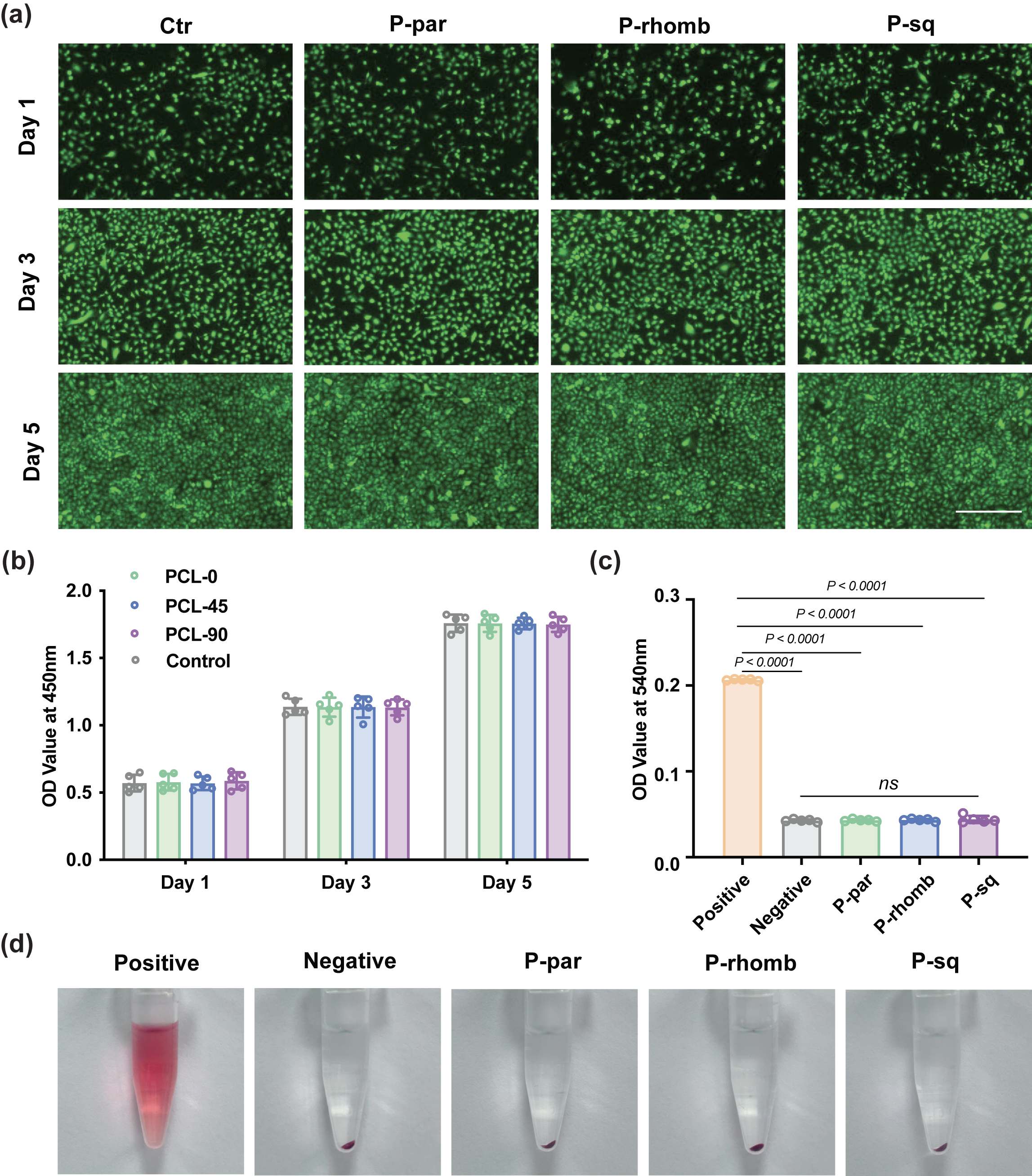

Supplement: Supplementary 1 — Figs. S1 to S3 Table S1 [file research.0499.f1.zip › Figure S2.tif]

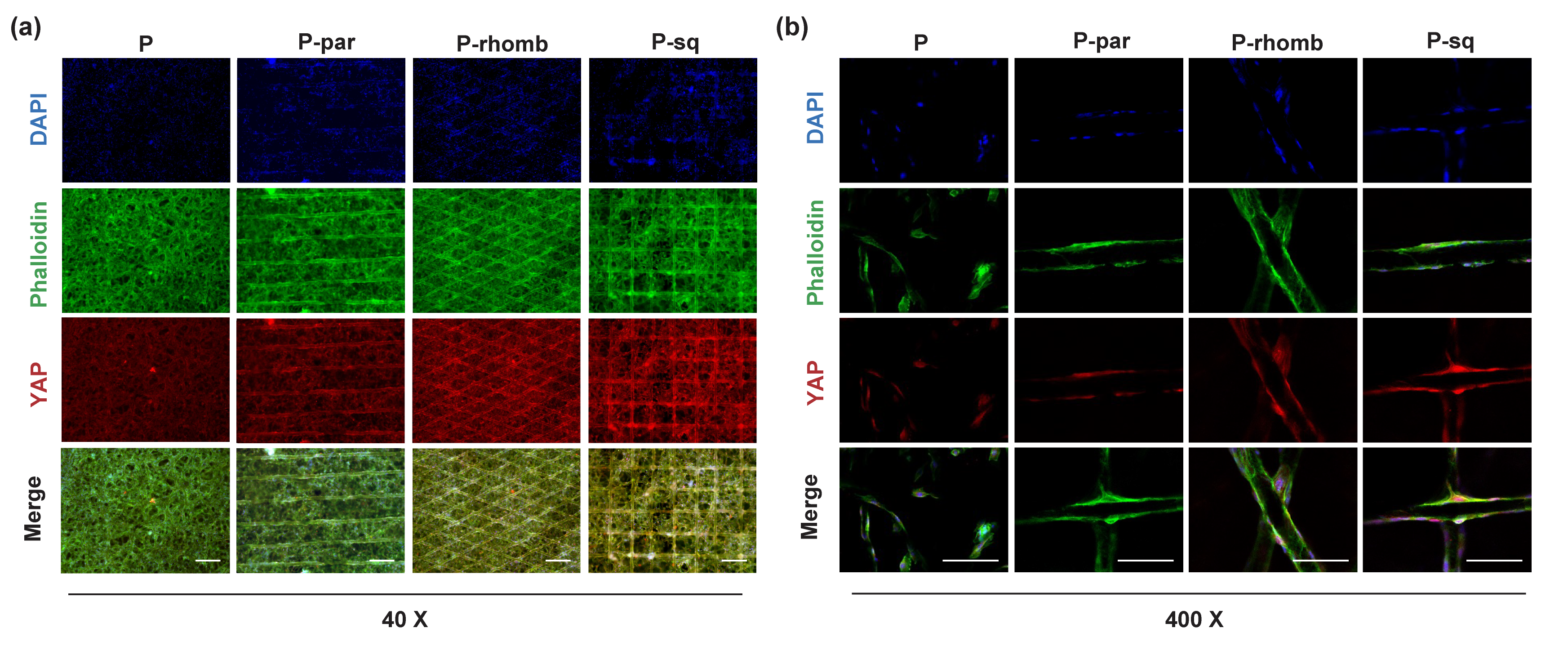

Supplement: Supplementary 1 — Figs. S1 to S3 Table S1 [file research.0499.f1.zip › Figure S3.tif]
